# Soil Contamination with Metals in Mountainous: A Case Study of Jaworzyna Krynicka in the Beskidy Mountains (Poland)

**DOI:** 10.3390/ijerph20065150

**Published:** 2023-03-15

**Authors:** Sławomir Dorocki, Joanna Korzeniowska

**Affiliations:** Institute of Geography, Pedagogical University of Krakow, 30-084 Krakow, Poland

**Keywords:** heavy metals, soil, absolute altitude, Jaworzyna Krynicka, contamination

## Abstract

The paper presents the content of six metals (Cd, Cr, Cu, Ni, Pb, and Zn) in the soils of the southern slope of Jaworzyna Krynicka in Poland. Soil samples were collected in polygons, starting from an altitude of 500 m above sea level and ending at an altitude of 1100 m above sea level. Ten soil samples were collected in each polygon. The polygons were set at every 100 m of absolute altitude. The selected research area is an important natural area. The fertile mountain beech forests located there are the most important forest communities in the mountain areas of Poland. They are valuable habitats for plants and animals (especially for large predatory mammals). Every year, numerous tourists and health resort patients visit this place. The results of the research showed that soil contamination in the study area is not high, in particular for altitudes of 500 and 900 m above sea level. At these altitudes, the contents of Cd, Cr, Cu, Ni, Pb, and Zn were similar to the concentrations of these metals in uncontaminated soils. The tests carried out showed very low cadmium content for all absolute altitudes. Zinc, the concentrations of which exceeded natural values, showed the highest content in the tested soils. All the metals tested showed a common tendency of increases in their content in the soils of Jaworzyna Krynicka up to 800 m above sea level. From an altitude of 900 m above sea level, the content of these metals decreased, except for Pb. Only Pb concentrations in Jaworzyna Krynicka soils also increased with the increasing altitude. The research significance of this work is that it is important for assessing the ecological balance in the selected area.

## 1. Introduction

Heavy metals are very common in the environment. Metals present in the form of salt solutions, which are by-products or wastes of various industries, pose a threat to the environment [1,2,3,4,5,6].

The condition for the sustainable existence of any ecosystem is the balance of the main metabolic processes, i.e., homeostasis. In natural ecosystems, biogeochemical cycles are usually characterized by a certain regularity. However, as a result of human economic activity, they may be disturbed owing to the excessive discharge of one or more components. The constant increase in the consumption of trace elements leads to changes in the proportion between their activation and introduction into the biological environment and their re-deposition in geological formations [3,7].

Dusts contained in the atmosphere, in which heavy metals are present, get into the soil and fall on the above-ground parts of plants. As a result, the concentration of heavy metals in these elements of the environment increases. The natural content of heavy metals in soils is closely related to the type, kind, and grade of soil [3,4,7]. The intensity of metal deposition depends on the emission volume, physical properties of dusts, meteorological conditions, and soil characteristics.

Five metals (Cd, Cr, Cu, Pb, and Zn) from the group of elements with a very high degree of threat to the natural environment and one (Ni) with an average degree of threat were selected for the study. Introduced into the soil, these metals accumulate in it and remain there for a long time [8,9,10]. Heavy metals are often transported over long distances from emission sources [11,12,13]. This phenomenon is related to the long-term dustiness of the atmosphere and meteorological conditions. The residence time of particulate matter in the atmosphere also depends on the size of the particles and the configuration of the terrain onto which they fall. Low pressure and strong winds favor the spread of pollutants over long distances [14,15].

The aim of this study was to determine the content of Cd, Cr, Cu, Ni, Pb, and Zn in the soil, at various absolute altitudes, of an environmentally important area, the Krynica-Zdrój and Muszyna health resorts located in the Poprad Landscape Park. Krynica-Zdrój, as well as Muszyna, which borders it, are towns with the status of health resorts, which are visited by many tourists and health resort patients [16,17]. These towns are so popular owing to their healing waters, climatic conditions, and extensive tourist and skiing infrastructure [18,19]. It is an area located in the Beskid Sądecki. The object of the research was the area of the southern slope of Jaworzyna Krynicka, reaching an altitude of 1114 m above sea level. This peak is located on the border of the two mentioned health resorts; however, the samples were taken from the area administratively belonging to the Muszyna commune. The research work carried out is important for determining the impact of long-range emissions on the natural environment of mountain areas. The increased content of heavy metals in the soils of environmentally valuable areas clearly indicates an anthropogenic source of pollution.

## 2. Materials and Methods

### 2.1. Study Area

Soil samples were collected on the southern slope of Jaworzyna Krynicka, which is the highest peak (1114 m above sea level) in the Jaworzyna Krynicka range in the Beskid Sądecki, Poland. The Jaworzyna Krynicka range occurs within the Magura Nappe. The spatially dominant geological formations within the range are thick-bedded Magura sandstones [20]. The sandstones are represented by psamite–pelite deposits, conglomerated with a clay or lime–clay binder. They result in a rock mantle with a mechanical composition of light and medium loams, sometimes dusty, with a skeleton of various sizes, occurring at a depth of 40–50 cm, and sometimes shallower [21]. 

Climatic conditions in the mountainous area of the Carpathians are difficult to determine. Their parameters depend on numerous factors, such as altitude above sea level, exposure, lie of the land, etc. The climate of the study area is closely related to the orographic location and massiveness of the culminating part of the Jaworzyna Krynicka range. The conditions prevailing there correspond to the habitat types of forests, in particular to the growth and development of mountain forests and mountain mixed forests. In the case of advective weather, situations with air inflow from the west (18.8%) as well as from the south and north-west prevailed, on average, in 11.0% of all cases. These situations generally prevailed in all months, with the period of their greatest activity being the cold part of the year, from October to March. In the summer (August and September), baric systems with air advection from the east and north-east prevailed [22].

The forests of Jaworzyna Krynicka consist mainly of a mixed mountain forest, located mainly above an altitude of 950 m above sea level, and a mountain forest. In the study area, beech accounted for about half of the stand. Apart from beech, there are also spruce, fir, and pine trees. The role of admixture species is played here by larch, birch, and grey alder, while ash and sycamore trees also occur, but singly [23].

### 2.2. Sampling

**Figure 1:**
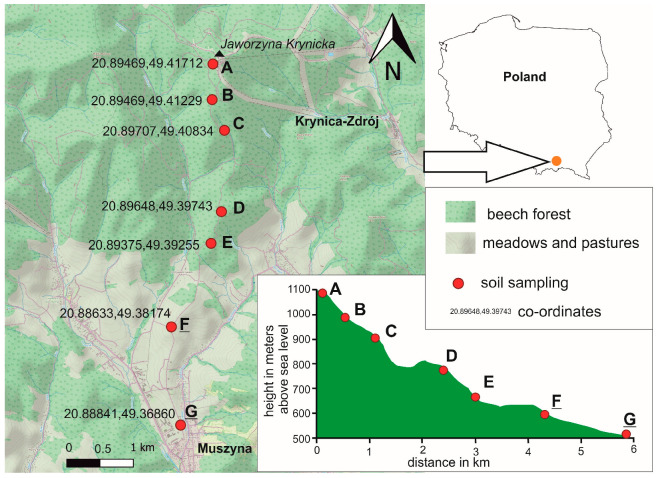
Location of sampling sites.

Samples (ten samples per polygon) were taken from seven polygons (A, B, C, D, E, F, and G) at altitudes of 1100, 1000, 900, 800, 700, 600, and 500 m above sea level, located in an area with southern exposure (Figure 1). The samples were taken on 24 May 2021. The polygons were distributed along the hill stretching between the Szczawniczek stream in the west and the Jastrzębik stream in the east, and descending from the top of Jaworzyna towards the village of Złockie in the Muszyna-Zdrój commune. 

### 2.3. Chemical Analysis

In order to determine the content of heavy metals (Cd, Cr, Cu, Ni, Pb, and Zn) in the sampled soil material (topsoil, up to 10 cm), the following laboratory work was carried out, in accordance with the methodology used for collecting and preparing samples for chemical analyses [24,25]:

- Manually cleaning the collected samples by removing foreign material (dry leaves, twigs, grass, etc.);
- Drying the samples at 100 °C;
- Grinding soil samples in a ceramic mortar and sieving through a sieve with a mesh diameter of 2 mm;
- Mineralization, which is performed to completely break down soil samples into simple, solid compounds—1 g of the dried sample material was digested with a modified Aqua Regia solution of equal parts concentrated HCl, HNO_3_, and DIH2O for one hour in a heating block or hot water bath. The resulting solution was filtered and stored in sealed polyethylene containers until sent for spectrometric analysis;
- Determination of the pseudo-total content of heavy metals using the inductively coupled plasma mass spectrometry (ICP-MS) method in the Bureau Veritas Commodities, Vancouver BC, Canada. The use of the Bureau Veritas methodology made it possible to accurately determine the metal content in the soil material, with the following detection limits (mg/kg) for Cd: 0.01, Cr: 0.5, Cu: 0.01, Ni: 0.1, Pb: 0.01, and Zn: 0.1. The STD DS11 and STD OREAS262 standards were used as reference materials.

### 2.4. Statistical Analysis of the Data

SAS^®^ OnDemand for Academics software and ANOVA procedures were used for statistical analysis [26]. The software SAS^®^ OnDemand for Academics is available on the website https://www.sas.com belonging to SAS Institute Inc. (SAS Campus Drive, Cary, NC, USA).

The ANOVA procedure was used to analyse the content of selected elements in the soil by performing the analysis of variance [27]. In the analysis of variance, the continuous response variable, considered as the dependent variable, is measured using classification variables, called independent variables. It was assumed that the variability of the obtained results follows from the adopted classification and depends on the altitude above sea level of the collected soil sample, with random error responsible for the remaining variability. The classification variable was specified in the code by the CLASS value which, unlike the GLM procedure, does not allow continuous variables on the right side of the model.

The F test indicates whether the model takes into account a sufficient amount of variability of the dependent variable [28]. The general F test is statistically significant in all examined cases of the analyzed elements, which indicates that the adopted model for a given element, as a whole, is responsible for a significant part of the variability of the dependent variable. The F test indicates that there is a difference between the mean values for individual samples according to the altitude above sea level. Interaction between the altitude of a collected soil sample and the content of selected elements in the soil is significant at a level of 95%. The significance level of this test was determined prior to the analysis. The F test does not reveal any information about the nature of the observed differences between the content of elements and altitude. Therefore, mean comparison methods were used to collect further information. The MEANS instruction requires the comparison of average levels using the Waller–Duncan K-ratio test method [29]. The Waller–Duncan K-ratio test is a multi-range test. It does not work by controlling for type I errors. Instead, it compares type I and type II error rates based on Bayesian principles [30].

In addition, the analysis used some simple statistics. The value of the R-square relationship indicates how much the model of regression between the content of metals in the soil and altitude corresponds to the variability of the variable [31]. The coefficient of variation (CV) is a measure determining the diversity of the analyzed samples together. The CV shows the degree of variation of the data in the sample relative to the mean population, even if they differ in the mean value. The mean square error (MSE) also provides information about how close the regression line is to the set of points, i.e., it proves the variability of the data in the same way as the mean of the dependent variable [32]. Root MSE (RMSE) is an estimate of the standard deviation of the dependent variable [33]. RMSE is one of the most commonly used measures to assess the quality of forecasts. It shows how far predictions deviate from the actual values measured using the Euclidean distance. In the case below, it refers to the model of regression between metal content in the soil.

## 3. Results

Among the analyzed elements, the best match of the model of regression according to the altitude of sampling and the content of elements in the soil was noted for Ni and Cr, where the R-Square was 0.971 and 0.963, respectively. Only in the case of Cd, the R-Square was below 0.9 and reached the value of 0.723, which is already a high value. 

The results obtained for individual elements show that in most cases, the model of regression corresponds to the variability of the variable (Table 1).

Regarding the variability of the results obtained on the basis of the coefficient of variation (CV), the smallest diversity was noted among the tested elements in the case of Cr (7.6). Low values in the samples taken also occurred for Ni and Zn (around 10.8). The greatest diversity was noted in the case of Cd, which was mainly the result of two samples taken at altitudes of 1000 and 1100 m above sea level. However, in no case were the values such outliers as to be omitted from the analysis.

Considering the root mean square error (RMSE), or the square mean of errors, which is the square root of the MSE, it can be observed that the smallest difference between the obtained estimation from the model and the actual value occurs in the case of Cd (0.091). Low values of the difference between the values obtained from the model and the actual values were noted for Cr (1709), Ni (2383), and Cu (2736). In other cases, the value of the standardized difference is higher and, in the case of Zn, it is as much as 8 points.

Additionally, in the case of the mean square error (MSE) for the grouping model, the smallest difference between the model and the observed values was noted for Cd content (0.008) and, in ascending order, for Cr (2.921), Ni (5.677), and Cu (7.483). The greatest differences were noted, as before, for Zn (64.314).

**Figure 2:**
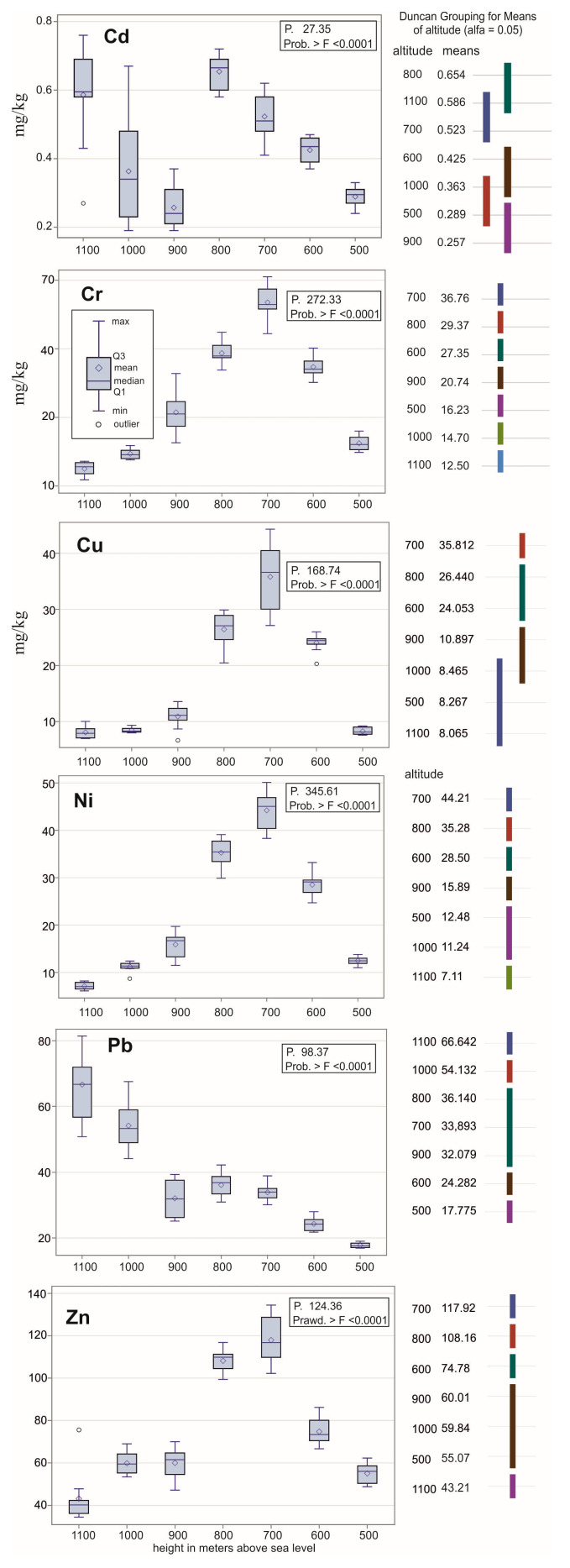
The content of metals in the soil according to altitude and the grouping of polygons according to the average content of elements in the soil.

Therefore, referring to the distribution of elements in the soil samples taken at specific altitudes, in the case of Cr, Cu, Ni, and Zn, the metal content increases from 500 m above sea level to 700 m above sea level, and then it usually drops below the value from 500 m above sea level (Figure 2). The change in metal content may, on the one hand, result from the altitude above sea level, but also from the type of land cover (the two lowest polygons were without any forest). 

In the case of Cd, the concentration of metals increases from an altitude of 500 to 800 m above sea level, and then at 900 m, for example, it decreases to the level from 500 m above sea level, to reach at an altitude of 1100 m above sea level the same value as at 800 m above sea level.

In the case of Pb content, the values show a slow increase from an altitude of 500 to 900 m above sea level. Samples from altitudes of 700 to 800 m above sea level have the same average at a level of 0.05. On the other hand, samples from the highest polygons have the highest values of Pb content.

In the analysis of grouping according to average levels using the Waller–Duncan K-ratio coefficient method, polygons that are graphically connected by the same line have the same average value at a confidence level of 0.05. All samples have a significantly different average across all polygons only in the case of Cr. In the case of Cd and Cu, there are the smallest differences in the average values according to the altitude of sampling.

## 4. Discussion

The first polygon (A) at an altitude of 1100 m above sea level was located below the tourist-developed peak of Jaworzyna Krynicka, overgrown mainly with beech, with blueberry with an admixture of spruce included in the meso-climatic floor of the peak area. As in the case of the polygons from A to E, there were cryptopodzolic soils or brown acidic soils. Additionally, in the case of samples from polygons B and C, these were beech forest habitats. The reaction of these soils from polygons A, B, and C was very acidic (pH in H_2_O) and varies in a range of 4.0–4.3. In polygons D and E, mixed beech–spruce–pine stands in a mountain forest habitat have a greater share. The reaction of these soils was already acidic and slightly acidic, in a range of 5.5–6.3. Polygon F was grassland. It represents a grassy habitat with the predominance of fescue, bent grass, and dog’s-tail grass. The grassland was characterized by a reaction close to neutral or slightly acidic (pH ranging from 6.5 to 6.9). Therefore, these were proper brown soils and acidic soils. Polygon G, located at the lowest level and surrounded by buildings located in adjacent valleys, was an agricultural area used as pastures and farmland. These were mainly leached brown soils. These soils, similar to the soils in polygon F, had a neutral and slightly acid reaction (6.2–7.2). The organic carbon content in the soils increased with altitude, starting from 2.38% (500 m above sea level), through 2.91% (600 m above sea level), then 6.53% (700 m above sea level), 7.45% (800 m above sea level), and then 9.12% (900 m above sea level), reaching 10.42% at 1000 m, and 12.34% at 1100 m above sea level. [21]. The average content of the tested metals in the soils of Jaworzyna Krynicka within each polygon was compared with the globally defined geochemical background (average content of the element in the Earth’s crust according to Turkian and Wedepohl [34]) and with the value of the locally defined geochemical background for Polish soils proposed by Czarnowska [35]. The average content of the element in the Earth’s crust [34] and the geochemical background for Polish soils [35] amount to, respectively: for Cd, 0.18 and 0.13 mg/kg; for Cr, 27.0 and 13.0 mg/kg; for Cu, 7.1 and 14.0 mg/kg; for Ni, 10.2 and 15.0 mg/kg; for Pb, 9.8 and 17.5 mg/kg; for Zn, 30.0 and 50 mg/kg.

The contents of Cu, Ni, Pb, and Zn are almost twice as high in Polish soils as in world soils. However, the contents of Cd and Cr are lower in Polish soils.

The average contents of Cd and Pb (for all absolute altitudes) in the soils of the southern slope of Jaworzyna Krynicka are higher than both the globally defined geochemical background (average content of the element in the Earth’s crust) and the geochemical background for Polish soils. The average contents of Zn and Cu in the tested soils are higher than the globally defined geochemical background for all tested altitudes, while for altitudes of 500–1000 m above sea level, the Zn content is higher than the geochemical background for Polish soils. The average Cu content is higher than the geochemical background for Polish soils only for an altitude of 600–800 m above sea level. The average Ni content in the soils of Jaworzyna Krynicka is higher, for all altitudes except 1100 m above sea level, than the globally defined geochemical background, and for altitudes of 600–900 m above sea level, higher than the geochemical background value for Polish soils. The average Cr content is higher than the globally defined geochemical background only for altitudes of 700 and 800 m above sea level, while the tested Cr content is higher, for all altitudes except 1100 m above sea level, than the geochemical background for Polish soils.

One should consider why the concentrations of all metals tested, except for Pb, increase up to an altitude of 800 m above sea level, and then decrease. Why do metal concentrations reach the highest values for altitudes of 700 and 800 m above sea level? Why do soil Pb concentrations increase rather than decrease with increasing altitude? The logical explanation seems to be the land cover and soil reaction. In the polygons located at altitudes of 1100, 1000, and 900 m above sea level, we are dealing with beech forest habitats, while in the polygons located at altitudes of 800 and 700 m above sea level, mixed stands of beech, spruce, and pine predominate in the mountain forest habitat. Here we can see the influence of conifer needles on the pH of the soil, which is definitely more acidic compared to higher altitudes. The polygons at altitudes of 600 and 500 m above sea level are grasslands, pastures, and farmland. Neutral to slightly acidic reaction prevails here. Moving on to the increase in Pb content in the soil of the slope of Jaworzyna Krynicka with increasing absolute altitude, this result of the study can be explained by the strong affinity of Pb with the content of organic matter in the soil. Organic matter (content of organic carbon) in the soil in the analyzed area increased, similarly to the concentration of Pb, with the altitude above sea level.

Comparing the results obtained with the metal content in the soils of environmentally important and slightly polluted areas, a similar content of Cr and Pb in the soils reported by Słowik et al. [36] for the Roztocze National Park was found. However, the content of Zn was almost twice as high as that measured for Jaworzyna Krynicka. Most likely, such a high content of Zn was affected by the type of soil found in the park. Similar concentrations of Cd, Cr, Cu, and Ni were also found in the soils of Jaworzyna Krynicka, and in the soils of the Stołowe Mountains National Park [37], and in the soils of the Tatra National Park [38]. The content of Pb and Zn in the Jaworzyna Krynicka soils was almost twice as high as the content of this metal in the soils of the above-mentioned parks.

The results of the biomonitoring of atmospheric aerosol pollution are often interpreted using the enrichment factor (EF) [39,40,41]. However, the best results are obtained when comparing the content of metals in soil and mosses. Therefore, it was not used in this case. However, further research is planned to compare the content of the tested metals in soil and mosses.

## 5. Conclusions

The mean contents of all the tested metals in the soils of the southern slope of Jaworzyna Krynicka, for altitudes of 500 and 900 m above sea level were within the range of values natural in Polish soils. Cadmium was the metal which did not exceed natural levels in soils for all absolute altitudes. The metal which, for all the tested altitudes, exceeded the values natural in Polish soils was zinc. 

The mean concentrations of metals for altitudes of 700 and 800 m above sea level were highest for Cd, Cr, Cu, Ni, and Zn. An increase in the content of these metals was observed from an altitude of 500 to 800 m above sea level, and then a decrease in concentration began from 900 to 1100 m above sea level. The Pb content increased with the increase in absolute altitude, reaching maximum values for an altitude of 1100 m above sea level.

## Figures and Tables

**Table 1:** The results of the statistics of the content of individual metal elements in the soil according to the variable altitude above sea level.

Statistical Indicators	Cd	Cr	Cu	Ni	Pb	Zn
R-Squared	0.72	0.96	0.94	0.97	0.90	0.92
Coefficient of variation (CV)	20.61	7.59	15.70	10.78	14.32	10.82
Root mean square error (RMSE)	0.091	1.709	2.736	2.383	5.419	8.020
Mean square (MS)	0.22	795.52	1262.76	1962.12	2888.44	7998.22
F value (<0.0001)	27.35	272.33	168.74	345.61	98.37	124.36
Mean square error (MSE) of grouping model	0.008	2.921	7.483	5.677	29.362	64.314

## Data Availability

The data presented in this study are available on request from the corresponding author. The data are not publicly available due to its further use for comparative research.
